# Newly Recognized Spotted Fever Group *Rickettsia* as Cause of Severe Rocky Mountain Spotted Fever–Like Illness, Northern California, USA

**DOI:** 10.3201/eid3007.231771

**Published:** 2024-07

**Authors:** Will S. Probert, Monica P. Haw, Aran C. Nichol, Carol A. Glaser, Sarah Y. Park, Laura E. Campbell, Kavita K. Trivedi, Hannah Romo, Megan E.M. Saunders, Anne M. Kjemtrup, Kerry A. Padgett, Jill K. Hacker

**Affiliations:** California Department of Public Health, Richmond, California, USA (W.S. Probert, M.P. Haw, C.A. Glaser, H. Romo, M.E.M Saunders, K.A. Padgett, J.K. Hacker);; Kaiser Permanente, Oakland, California, USA (A.C. Nichol); Karius, Redwood City, California, USA (S.Y. Park);; Alameda County Public Health Department, San Leandro, California, USA (L.E. Campbell, K.K. Trivedi);; California Department of Public Health, Sacramento, California, USA (A.M. Kjemtrup)

**Keywords:** vector-borne infections, bacteria, spotted fever group Rickettsia, Rickettsiosis, Rocky Mountain spotted fever, ticks, real-time PCR, multilocus sequence typing, California, United States

## Abstract

The incidence of spotted fever group (SFG) rickettsioses in the United States has tripled since 2010. Rocky Mountain spotted fever, the most severe SFG rickettsiosis, is caused by *Rickettsia rickettsii*. The lack of species-specific confirmatory testing obfuscates the relative contribution of *R. rickettsii* and other SFG *Rickettsia* to this increase. We report a newly recognized rickettsial pathogen, *Rickettsia* sp. CA6269, as the cause of severe Rocky Mountain spotted fever–like illness in 2 case-patients residing in northern California. Multilocus sequence typing supported the recognition of this pathogen as a novel *Rickettsia* genotype most closely related to *R. rickettsii*. Cross-reactivity observed for an established molecular diagnostic test indicated that *Rickettsia* sp. CA6269 might be misidentified as *R. rickettsii*. We developed a *Rickettsia* sp. CA6269–specific real-time PCR to help resolve this diagnostic challenge and better characterize the spectrum of clinical disease and ecologic epidemiology of this pathogen.

Rickettsioses are undifferentiated febrile illnesses, often accompanied by myalgia and rash, that are caused by intracellular gram-negative bacteria of the genus *Rickettsia*. Spotted fever group (SFG) *Rickettsia* are transmitted through the bite of ticks or mites and are grouped together on the basis of antigenic and genetic similarities that distinguish this group from other *Rickettsia*, namely the typhus group. The incidence of SFG rickettsioses in the United States increased 3-fold from 2010 to 2018 (*1*). The SFG *Rickettsia* associated with human illness in the United States include the tickborne agents *R. rickettsii* (Rocky Mountain spotted fever [RMSF]), *R. parkeri* (*Rickettsia parkeri* rickettsiosis), and *Rickettsia* 364D (Pacific Coast tick fever [PCTF]) and the miteborne agent *R. akari* (rickettsialpox) (*1*,*2*). Among the SFG rickettsioses, RMSF is the most severe; its case-fatality rate is 5%–10% in the United States (*3*,*4*). Unfortunately, the relative contribution of *R. rickettsii* and other rickettsial agents to the increase in SFG rickettsioses is largely unknown because of the reliance on group-specific (SFG or typhus group) serologic testing and the lack of species-specific confirmatory testing (*1*,*5*).

In California, SFG rickettsioses are a reportable condition tracked for public health surveillance purposes. California has the lowest incidence of SFG rickettsioses among reporting states and averages only 11 probable and 1 confirmed SFG rickettsiosis cases annually (*1*,*6*,*7*). Only RMSF and PCTF are endemic to California (*7*). Ticks infected with *R. rickettsii* are extremely rare in California; only 1 infected *Dermacentor occidentalis* tick and 2 *Rhipicephalus sanguineus* ticks have been detected despite numerous environmental surveys (*7*). In contrast, *Rickettsia* 364D, a genetic near neighbor of *R. rickettsii*, is found in 2.1% of adult *D. occidentalis* ticks (*8*,*9*). PCTF was recognized in 2008 and is clinically less severe than RMSF (*10*). The presence of an inoculation eschar at the site of a tick bite and lack of a rash can be useful in differentiating PCTF from RMSF (*9*). Another genetic near neighbor of *R. rickettsii*, *Rickettsia* sp. CA6269, recently was reported in rabbit ticks (*Haemaphysalis leporispalustris*) collected in Northern California (*11*). Multilocus sequence typing (MLST) of that strain revealed a novel genotype having sufficient sequence divergence from *R. rickettsii* to lead the investigators to propose a new species, *Candidatus* Rickettsia lanei. Here, we describe the clinical and epidemiologic features of severe RMSF-like illness in 2 patients in northern California who had disease onset dates separated by nearly 20 years and whose illness was caused by a newly identified SFG *Rickettsia*, *Rickettsia* sp. CA6269. 

## Materials And Methods

### Clinical Specimens

Clinical specimens for confirmatory testing of suspected rickettsial diseases were submitted to the California Department of Public Health Viral and Rickettsial Disease Laboratory (CDPH-VRDL) for public health surveillance purposes and considered exempt from human subject regulations by the California Health and Human Services Agency Committee for the Protection of Human Subjects (project no. 2023-085). In addition to a plasma specimen from the initial case-patient, archival clinical specimens (serum or plasma) collected over the past 20 years from 8 confirmed SFG rickettsiosis case-patients were available for molecular characterization. SFG rickettsiosis cases were classified as confirmed for public health surveillance purposes on the basis of clinical and laboratory criteria (*12*).

### Nucleic Acid Extraction and Real-time PCR 

We extracted total nucleic acids using the NucliSENS easyMAG instrument (bioMérieux, https://www.biomerieux.com) with a serum or plasma input volume of 300 µL and a total nucleic acids output volume of 110 µL. We further concentrated total nucleic acids from case-patient 2 by 4-fold using the RNA Clean and Concentrator Kit (Zymo Research, https://www.zymoresearch.com).

We tested specimens for *Rickettsia* using a laboratory-developed triplex real-time reverse transcription PCR (rRT-PCR) targeting a *R. rickettsii*–specific 23S rRNA single-nucleotide polymorphism (SNP), a *R. typhi*–specific 23S rRNA SNP, and genus-specific regions of the 23S rRNA (Appendix). The assay was developed and the performance specifications established by the CDPH-VRDL as required under the Clinical Laboratory Improvement Amendments. A *R. rickettsii*–specific real-time PCR (rPCR) assay, RRi6, was performed as described by Kato et al. (*13*); the assay does not detect *Rickettsia* 364D (*14*).

### MLST

We amplified and sequenced 6 target sequences, 23S rRNA, 16S rRNA, *gltA*, *ompA*, *ompB*, and *sca4* (Appendix); the last 5 of those targets correspond to the regions recommended for the gene sequence–based classification of *Rickettsia* (*15*). A commercial laboratory performed bidirectional Sanger sequencing (ELIM Biopharmaceuticals, https://elimbio.com). We performed sequence editing and assembly using Geneious Prime 2022.0.2 (https://www.geneious.com). Nucleotide BLAST searches of the National Center for Biotechnology Information (NCBI) nucleotide collection and whole-genome shotgun contigs databases (https://blast.ncbi.nlm.nih.gov) were performed to identify genetic near neighbors and facilitate phylogenetic analyses. We concatenated and aligned the 16S rRNA, *gltA*, *ompA*, *ompB*, and *sca4* sequences for validly named SFG *Rickettsia* species and constructed a maximum-likelihood phylogenetic tree using MEGA 10 (https://www.megasoftware.net). We measured the reliability of the resulting tree by bootstrap resampling using 1,000 replicates.

### Tick Collection and Testing

We conducted field investigations at potential exposure sites by flagging vegetation and leaf litter for ticks. We identified tick species, sex, and life stages by examining morphologic features. For the 2023 case investigation, we processed adult ticks collected from Alameda and Contra Costa Counties for nucleic acid extraction using the DNeasy Blood and Tissue Kit (QIAGEN, https://www.qiagen.com). We screened tick nucleic acid extracts for *R. rickettsii* using the RRi6 assay. Ticks collected and identified from San Mateo and Marin Counties during the 2004 case investigation were screened for *R. rickettsii* DNA by the US Army Center for Health Promotion and Preventative Medicine–West.

### Design and Development of a *Rickettsia* sp. CA6269 Real-Time PCR 

We performed a nucleotide BLAST search using the *ompA* sequence from *Rickettsia* sp. CA6269 (GenBank accession no. JN990595) and downloaded and aligned selected sequences using the multiple sequence alignment tool. We selected regions of sequence divergence for primer and probe design using Primer-BLAST (https://www.ncbi.nlm.nih.gov/tools/primer-blast). We designed a 5′ exonuclease rPCR to amplify and detect a 146 bp region of *ompA*. The assay oligonucleotides were synthesized by Integrated DNA Technologies (https://www.idtdna.com). The 25-µL reaction mixture consisted of RLompAF1 (5′-GGGCACTTGGTGTTCCTACA-3′) and RLompAR1 (5′-AAATGCCCAATTGTTTTGAGGAC-3′) primers at 500 nM, RLompAP1 (6FAM- CTAATGGTG/ZEN/ATCCTACTGGCG-3IABkFQ) probe at 100 nM, 1X Premix ExTaq (Probe qPCR) mix (Takara Biosciences, https://www.takarabio.com), and 5 µL of total nucleic acids. We performed amplification and fluorescence detection with the ABI 7500 FAST DX Sequence Detection System (ThermoFisher Scientific, https://www.thermofisher.com) using the fast mode and the following amplification conditions: 1 minute at 95°C, 45 cycles of 95°C for 3 seconds, and 60°C for 30 seconds. We assessed assay analytical specificity by using a panel of 37 total nucleic acid extracts from members of the order Rickettsiales (Appendix) and evaluated analytical sensitivity by using 10-fold serial dilutions of a quantified 156-bp gBlock gene fragment (Integrated DNA Technologies) spiked into nucleic acids from pooled human blood and tested in replicates of five. We defined the assay limit of detection as the smallest number of DNA copies detected per reaction for all 5 replicates.

## Results

### Case-Patient 1

The CDPH was notified of a suspected RMSF case involving a man with symptom onset in July 2023. The case-patient sought care at the emergency department (ED) with a 3-day history of influenza-like symptoms; he experienced fever, headaches, myalgias, arthralgias, malaise, loss of appetite, nonbloody diarrhea, and left-sided abdominal pain. Vital signs included temperature of 103°F (39.4°C), pulse of 96 beats/min, blood pressure of 116/71 mm Hg, and respiratory rate of 16 breaths/min. Lower left quadrant and suprapubic abdominal tenderness were noted upon physical examination. Of note, a rash was not observed. An antimicrobial regimen of ceftriaxone, metronidazole, and oral vancomycin was initiated, and the case-patient was hospitalized. The case-patient was transferred to the intensive care unit (ICU) on day 3 of hospitalization because of worsening hypoxemia, acidosis, encephalopathy, and seizures. Doxycycline was added to the treatment regimen on day 3 of hospitalization after an infectious diseases consultation that included rickettsial diseases in the differential diagnosis because of the severity of illness and clinical manifestations (Table 1).

**Table 1:** Clinical and laboratory features of *Rickettsia* sp. CA6269 infections in study of newly recognized SFG *Rickettsia* as cause of severe RMSF-like illness, northern California, USA*

Feature	Case-patient 1	Case-patient 2
Clinical		
Fever at hospital admission	Present, 103°F (39.4°C)	Present, 101.8°F (38.8°C)
Inoculation eschar	Absent	Absent
Rash	Absent	Present, maculopapular including palms
Myalgia	Present	Present
Headache	Present	Present
Nausea	Present	Present
Vomiting	Present	Present
Diarrhea	Present	Absent
Abdominal pain	Present	Absent
Acute kidney injury	Present	Absent
Photophobia	Present	Present
Altered mental status	Present	Present
Acute respiratory failure	Present	Present
Cutaneous necrosis and gangrene	Present	Absent
Coma	Present	Present
Days in intensive care unit	11	4
Days hospitalized	22	13
Laboratory†		
Serum sodium	Low (124 mEq/L)	Low (133 mEq/L)
Serum lactate dehydrogenase	High (421 U/L)	High (1,280 U/L)
Serum creatinine	High (1.44 mg/dL)	Unremarkable (1.0 mg/dL)
Serum bilirubin, total	High (2.3 mg/dL)	Unremarkable (0.6 mg/dL)
Aspartate transaminase	High (483 U/L)	High (268 U/L)
Alanine transaminase	High (323 U/L)	High (125 U/L)
CBC platelets	Low (45K/uL)	Low (80K/uL)
CBC leukocyte count	Unremarkable (4.5K/uL)	High (12.9K/uL)
CBC neutrophil percentage	Unremarkable (64%)	High (84%)
CSF glucose	NA	Unremarkable (69 mg/uL)
CSF protein	NA	High (147 mg/dL)
CSF leukocyte count	NA	High (9/uL)
SFG *Rickettsia* IgG titer (acute)	Unremarkable <1:128	Elevated (1:4,096)
SFG *Rickettsia* IgG titer (convalescent)	Elevated (>1:256)	Elevated (1:16,384)

*CBC, complete blood count; CSF, cerebral spinal fluid; NA, not applicable; RMSF, Rocky Mountain spotted fever; SFG, spotted fever group. †Samples for the blood metabolic panel, CBC, and CSF analyses (case-patient 2 only) were collected on the day of hospital admission for case-patient 1 and 3 days after hospital admission for case-patient 2.

Serologic testing for SFG *Rickettsia* by a commercial laboratory failed to detect a significant IgG titer in an acute serum specimen collected 5 days after symptom onset (Table 1). All blood cultures were negative. A laboratory diagnosis of rickettsial infection was initially made with the Karius Test, a microbial cell–free DNA (mcfDNA) metagenomic sequencing method (*16*). Sequencing of a plasma specimen collected 7 days after the onset of symptoms determined that *R. rickettsii* detected at 254,523 DNA molecules/microliter and *R. slovaca* detected at 97,653 DNA molecules/microliter were the best matches in the Karius Test genomic reference database. Serologic testing of a convalescent serum specimen, collected 105 days after symptom onset, demonstrated a significant IgG titer of >1:256 for SFG *Rickettsia*. The case-patient was discharged after being hospitalized for 22 days, including 11 days in the ICU, with a primary diagnosis of RMSF and secondary diagnoses of septic shock, acute kidney injury caused by acute tubular necrosis requiring intermittent hemodialysis, acute hypoxemic respiratory failure, encephalopathy, seizures, diarrhea, hyponatremia, abnormal liver enzymes, thrombocytopenia, supraventricular tachycardia, atrial fibrillation, and gangrene involving multiple digits on both hands.

The residual plasma specimen from mcfDNA sequencing was provided to the CDPH-VRDL for species-specific confirmatory testing. We tested total nucleic acids extracted from this sample with the triplex rRT-PCR that detects a *Rickettsia*-specific sequence, an *R. rickettsii*–specific SNP, and an *R. typhi*–specific SNP in the 23S rRNA. Unexpectedly, only the *Rickettsia* species target, and not the *R. rickettsii*–specific SNP, was detected (cycle threshold [Ct] value 24.9) (Table 2). Subsequent testing with the RRi6 assay detected DNA at a Ct value of 30.5 (Table 2). To address the discordant *R. rickettsii* test results for the triplex rRT-PCR and the RRi6 assay, we sequenced a 1,468-nt segment of the rickettsial 23S rRNA (GenBank accession no. OR600926). Comparative sequence analysis revealed a 2-nt difference from *Rickettsia* 364D and a 3- to 4-nt difference from *R. rickettsii*, including an alternate SNP allele for the rRT-PCR SNP target. We further assessed species relatedness using a MLST scheme established for the classification of *Rickettsia* (*15*). We amplified and sequenced a 1,408 nt segment of 16S rRNA, a 1,051 nt segment of *gltA*, a 587 nt segment of *ompA*, a 4,849 nt segment of *ompB*, and a 2,944 nt segment of *sca4* (GenBank accession nos. OR600927 and OR596747–50). Searches of the NCBI nucleotide databases revealed perfect sequence matches with *gltA* (290 nt) and *sca4* (673 nt), a single nucleotide insertion/deletion difference in *ompA* (471 nt), and a single SNP difference in *ompB* (758 nt) from *Rickettsia* sp. CA6269 (GenBank accession nos. JN990594–7) (*11*). MLST results indicated that the strain, designated CA23RL1, was nearly identical to *Rickettsia* sp. CA6269. In contrast, CA23RL1 displayed significant sequence divergence from *R. rickettsii* and *Rickettsia* 364D at each MLST target (Table 3). Phylogenetic analysis of concatenated sequences from validly named SFG *Rickettsia* demonstrated that CA23RL1 occupied a distinct branch and was most closely related to *R. rickettsii* (Figure).

**Table 2:** Comparison of real-time PCR results for case-patient 1 and retrospective SFG rickettsiosis case-patients in study of newly recognized SFG *Rickettsia* as cause of severe RMSF-like illness, northern California, USA*

SFG rickettsiosis case-patient	Specimen type	Cycle threshold value, by test type				
		Triplex *Rickettsia* spp.	Triplex *R. rickettsii* SNP	Triplex *R. typhi* SNP	RRi6 *R. rickettsii*	*Rickettsia* sp. CA6269 *ompA*
1	Plasma	24.9	ND	ND	30.5	29.6
2	Serum	34.3	ND	ND	34.3	34.4
3	Plasma	23.3	25.2	ND	25.4	ND
4	Serum	30.3	27	ND	34	ND
5	Serum	32.8	30.2	ND	35	ND
6	Serum	32.2	32.8	ND	33.6	ND
7	Plasma	29.5	29.2	ND	33.3	ND
8	Serum	20.8	19.2	ND	25.1	ND
9	Plasma	28.6	27.3	ND	30.2	ND

*ND, not detected; RMSF, Rocky Mountain spotted fever; SFG, spotted fever group; SNP, single-nucleotide polymorphism.

**Table 3:** *Rickettsia* sp. CA23RL1 sequence similarity with *Rickettsia rickettsii* and *Rickettsia* 364D in study of newly recognized SFG *Rickettsia* as cause of severe RMSF-like illness, northern California, USA *

*Rickettsia* sp. CA23RL1 target sequence	*R. rickettsii* sequence similarity†	*Rickettsia* 364 sequence similarity‡
16S rRNA	99.7%	99.6%
*gltA*	99.2%	99.6%
*ompA*	96.8%	97.3%
*ompB*	98.6%	98.8%
*sca4*	98.6%	98.6%

*RMSF, Rocky Mountain spotted fever; SFG, spotted fever group. †*Rickettsia rickettsii* strain Sheila Smith, GenBank accession no. CP000848.1. ‡*Rickettsia* 364D, GenBank accession no. CP003308.1.

**Figure:**
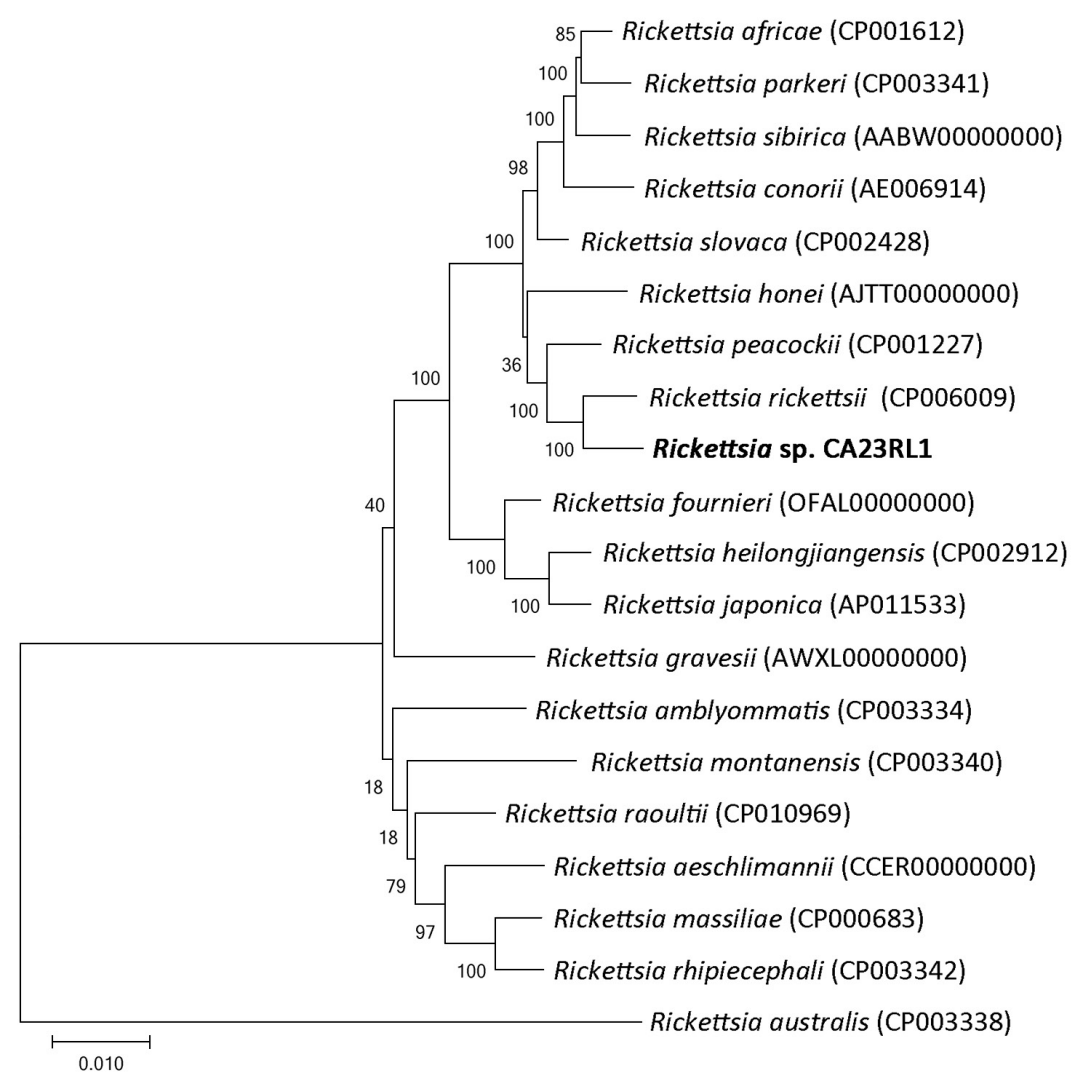
Maximum-likelihood phylogenetic tree of concatenated multilocus sequences in study of newly recognized spotted fever group *Rickettsia* as cause of severe Rocky Mountain spotted fever–like illness, northern California, USA. *Rickettsia* sp. CA23RL1 (bold text) occupies a distinct branch most closely related to *R. rickettsii*. *Rickettsia australis*, a transitional group *Rickettsia*, was included as an outgroup. Bootstrap values for 1,000 replicates are provided at each branch of the phylogenetic tree. GenBank accession numbers are provided in parentheses. Scale bar indicates evolutionary distance as measured by the number of substitutions per site.

The case-patient had not traveled outside of the San Francisco Bay area within 19 days of illness onset and did not recall a tick bite. The case-patient had played golf at 5 courses located in Alameda and Contra Costa Counties within 14 days of symptom onset, including the day symptoms began. Of note, the case-patient recalled entering vegetation to retrieve golf balls at several courses. Because that activity might have been a potential source of exposure to ticks, as follow-up we collected ticks from the 5 golf courses within 16–22 days (late July and early August) of illness onset. In all, 197 adult ticks were collected and identified as either *D. occidentalis* (n = 135) or *D. similis* (n = 62). None of the ticks were infected with *R. rickettsii* or *Rickettsia* sp. CA6269 as determined by the RRi6 assay.

### Case-Patient 2

We identified case-patient 2 through retrospective testing of 8 confirmed SFG rickettsiosis cases with the triplex rRT-PCR and RRi6 assays (Table 2). We detected *Rickettsia* spp. (Ct value 34.3), but not *R. rickettsii* or *R. typhi*, using the triplex rRT-PCR. The RRi6 assay detected rickettsial DNA (Ct value 34.3). Amplification and sequencing of a 397-nt segment of 23S rRNA (GenBank accession no. OR600925) by nested RT-PCR revealed an exact match to the 23S rRNA sequence determined for case-patient 1 and a 2- to 4-nt difference from *Rickettsia* 364D and *R. rickettsii*, supporting the identification of a second case of *Rickettsia* sp. CA6269 infection.

Case-patient 2, a male adult, was diagnosed with RMSF in 2004 on the basis of clinical features and a 4-fold increase in serologic titer to SFG *Rickettsia* in serum specimens collected 10 days apart (Table 1). Illness onset occurred in June; the case-patient sought care at the ED with a 5-day history of headaches, vomiting, photophobia, neck pain, and confusion. Upon initial examination, vital signs included a body temperature of 101.8°F (38.8°C), pulse of 96 beats/min, blood pressure of 143/89 mm Hg, and respiratory rate of 20 breaths/min. Physical examination revealed mild nuchal rigidity, a maculopapular rash on the arms and legs, and leg edema. The case-patient was hospitalized on the basis of the ED assessment that included encephalitis, sepsis, hypoxemia, rash of unknown etiology, and chronic leg edema, and antimicrobial treatment with ceftriaxone, vancomycin, and acyclovir was initiated. On day 3 of hospitalization, the patient became hypoxic and comatose and was intubated and transferred to the ICU for 4 days. Doxycycline was added to the treatment regimen on day 3 of hospitalization after an infectious diseases consultation that included rickettsioses in the differential diagnosis on the basis of clinical manifestations and the patient’s outdoor activities. Results of blood, CSF, urine, and sputum cultures were negative for significant pathogens. Serologic test results on day 10 of hospitalization indicated an IgG titer of 1:4,096 for SFG *Rickettsia*. After 13 days, the patient was discharged with a primary diagnosis of *Rickettsia* encephalitis.

The case-patient had not traveled outside the San Francisco Bay area during the 2 weeks before onset but had visited and camped at a county park and state beach in San Mateo County and a rural community in Marin County within that period. At the county park, he recalled finding a tick crawling on his body but not a tick bite. Field investigations at these locations conducted 30–40 days (July) after illness onset yielded 10 *D. occidentalis* ticks, 8 *D. similis* ticks, and 6 *Ixodes pacificus* ticks. Molecular testing of the *Dermacentor* spp. ticks did not detect *R. rickettsii*.

### *Rickettsia* sp. CA6269–Specific Real-time PCR 

After the sequence-based identification of *Rickettsia* sp. CA6269 infections, we developed an rPCR targeting genotype-specific regions of *ompA*. We did not observe assay cross-reactivity with nucleic acids from *Anaplasma phagocytophilum*, *Ehrlichia chaffeensis*, *Orientia tsutsugamushi*, and 14 *Rickettsia* species, including 10 *R. rickettsii* strains and 11 *Rickettsia* 364D strains (Appendix). The assay limit of detection was 1 copy of DNA per reaction. Of 9 SFG rickettsiosis case-patients tested, *Rickettsia* sp. CA6269 was detected only for case-patient 1 (Ct value 29.6) and case-patient 2 (Ct value 34.4) (Table 2). The remaining 7 case-patients were confirmed *R. rickettsii* infections on the basis of detection of the species-specific 23S rRNA SNP with the triplex rRT-PCR.

## Discussion

We describe the clinical and epidemiologic features of 2 case-patients with RMSF-like illness attributed to a newly recognized rickettsial pathogen, *Rickettsia* sp. CA6269. The case-patients experienced severe clinical manifestations shared with RMSF, including acute kidney injury and respiratory failure, cutaneous necrosis and gangrene, and encephalitis. No unique clinical features were recognized in the 2 case-patients that would distinguish between infections caused by *Rickettsia* sp. CA6269 and *R. rickettsii*. Both patients likely acquired the infections locally during outdoor activities: in 1 case, golfing at courses with adjacent wildlands, and in the other case, visiting parks and camping. Although neither case-patient recalled being bitten by a tick, 1 case-patient had observed a tick crawling on his body. Nearly half of reported RMSF cases do not recall tick bites (*4*). These cases illustrate that clinicians should remain cognizant of SFG rickettsiosis in the differential diagnosis of undifferentiated febrile illnesses despite the lack of travel to areas of rickettsiosis endemicity and the absence of a recognized tick bite. Field investigations at locations of suspected exposure produced mostly *Dermacentor* spp. ticks and no *H. leporispalustris* ticks. Molecular testing of *Dermacentor* spp. ticks, established vectors of *R. rickettsii*, failed to detect *R. rickettsii* or *Rickettsia* sp. CA6269. Those potential locations of exposure will be the focus of future environmental investigations timed to better correlate with peak *H. leporispalustris* tick questing behavior.

*Rickettsia* sp. CA6269 was discovered during a survey of *H. leporispalustris* ticks collected in California for SFG *Rickettsia* (*11*). Of 234 ticks collected at the northern California site, 1 larval pool and 1 nymph produced a unique genotype, *Rickettsia* sp. CA6269. This unique genotype was not identified in the 179 ticks collected in southern California. The investigators proposed designating this novel *Rickettsia* as *Candidatus* R. lanei on the basis of results from an abbreviated MLST scheme. We have extended this work by sequencing the full-length regions originally proposed for the sequence-based classification of *Rickettsia* and established that *Rickettsia* sp. CA6269 meets the 2003 criteria for defining a new *Rickettsia* species (*15*). More recently, 2 genome sequence-based methods have been proposed for the classification of *Rickettsia* species (*17*,*18*). One method used genome sequence–based criteria aligned with the classic taxonomic analyses of bacteria to delineate *Rickettsia* into 9 species; nearly all SFG *Rickettsia* were classified as a single species (*17*). The other approach used genome sequence–based criteria that account for the unique phenotypic characteristics of rickettsiae and established phylogenetic relationships that were consistent with the current taxonomic classification of *Rickettsia* (*18*). That method displayed a strong correlation with MLST for *Rickettsia* classification. Despite the lack of updated consensus criteria for the taxonomic classification of *Rickettsia* species, delineating *Rickettsia* sp. CA6269 as a strain or subspecies of *R. rickettsii* or a new species will benefit from isolating and cultivating strains, describing phenotypic characteristics including ecologic epidemiology, and determining whole-genome sequences.

Additional environmental studies are needed to investigate the geographic distribution, potential vectors, prevalence of infection, and reservoir hosts of *Rickettsia* sp. CA6269. The *H. leporispalustris* tick has been shown to be a maintenance vector of *R. rickettsii* and is distributed throughout the Americas from Alaska to Argentina (*19*,*20*). Adult *H. leporispalustris* ticks feed on rabbits and hares, whereas the nymph and larval stages feed on ground-frequenting birds and small rodents (*21*). Humans are rarely bitten by *H. leporispalustris*, indicating a limited role for this tick in transmitting SFG rickettsioses (*22*). Several surveys for *Rickettsia* infections in *H. leporispalustris* and rabbits have been conducted in California (*11*,*23*–*26*). In addition to the initial report of *Rickettsia* sp. CA6269, a further 2 studies have reported SFG *Rickettsia* detections in *H. leporispalustris*; 1 of those studies described the isolation of a *Rickettsia* antigenically related to *R. rickettsii* (*11*,*23*,*24*). Although likely rare, the case-patients described in this study might have encountered questing *H. leporispalustris* infected with *Rickettsia* sp. CA6269. Alternatively, *D. occidentalis*, a tick vector that more frequently bites humans and parasitizes a wide variety of mammals including lagomorphs, the preferred host of adult *H. leporispalustris*, might have been the source of *Rickettsia* sp. CA6269 transmission (*27*,*28*).

As with *R. rickettsii*, *Rickettsia* sp. CA6269 is likely a rarely encountered pathogen in California (*7*). After the initial detection of *Rickettsia* sp. CA6269, retrospective testing of 8 SFG rickettsiosis patient samples revealed a second case from nearly 20 years earlier. Nucleic acids from both cases lacked a *R. rickettsii*–specific 23S rRNA SNP but were detected with the RRi6 assay, indicating cross-reactivity of the RRi6 assay with *Rickettsia* sp. CA6269. The RRi6 assay was designed as *R. rickettsii*–specific on the basis of comparative genome analyses and targets a gene encoding a hypothetical protein absent in other rickettsial genomes including *Rickettsia* 364D (*13*,*14*). A homologue to this target sequence is likely present within the *Rickettsia* sp. CA6269 genome, creating the potential for misidentification of *Rickettsia* sp. CA6269 as *R. rickettsii* when using the RRi6 assay. To address this issue, we developed a *Rickettsia* sp. CA6269–specific rPCR assay that provides excellent analytical specificity and sensitivity. With more extensive performance characterization, this assay should prove useful for diagnostic testing and environmental screening of potential vectors and reservoir hosts of *Rickettsia* sp. CA6269.

In summary, we have identified *Rickettsia* sp. CA6269 as a causative agent of severe RMSF-like illness in California and have described an assay for its detection. The application of this new test, both prospectively and retrospectively, could identify additional cases, thereby leading to a better understanding of both the clinical spectrum of disease caused by *Rickettsia* sp. CA6269 and the relative contribution of this pathogen to the increasing incidence of SFG rickettsioses in the United States. In addition, the new test will enable environmental studies to define the ecologic epidemiology of this emerging pathogen.

Appendix Additional information about newly recognized spotted fever group *Rickettsia* as cause of severe Rocky Mountain spotted fever–like illness, northern California, USA.
