# Supplementary material for: Newly Recognized Spotted Fever Group Rickettsia as Cause of Severe Rocky Mountain Spotted Fever–Like Illness, Northern California, USA
Source: Emerg Infect Dis. 2024 Jul;30(7):1344–51. doi: 10.3201/eid3007.231771 (PMC11210658; doi:10.3201/eid3007.231771)
Supplement: Appendix — Additional information about newly recognized spotted fever group Rickettsia as cause of severe Rocky Mountain spotted fever–like illness, northern California, USA. [file 23-1771-Techapp-s1.pdf]

Article DOI: https://doi.org/10.3201/eid3007.231771

EID cannot ensure accessibility for supplementary materials supplied by authors. Readers who have difficulty accessing supplementary content should contact the authors for assistance.

Newly Recognized Spotted Fever Group *Rickettsia* as Cause of Severe Rocky Mountain Spotted Fever–Like Illness, Northern California

Appendix

Triplex Real-time Reverse Transcription PCR (rRT-PCR)

Specimens were tested for *Rickettsia* using a laboratory-developed triplex real-time reverse transcription PCR (rRT-PCR) assay targeting a *R. rickettsii*-specific 23S rRNA single nucleotide polymorphism (SNP) (T101C, GenBank accession number NR_103963), a *R. typhi*-specific 23S rRNA SNP (T1976C, GenBank accession no. NR_076209), and genus-specific regions of the 23S rRNA (Table S1). The assay was developed and validated by the California Department of Public Health Viral and Rickettsial Disease Laboratory (validation report available upon request). The rRT-PCR mixture consisted of 1X One Step PrimeScript III RT-PCR master mix (Takara Bio USA, www.takarabio.com/), Rrick23S_F and Rrick23S_R primers at 300 nM, Rrick23S_P probe at 120 nM, Rtyp23S_F and Rtyp23S_R primers at 500 nM, Rtyp23S_P probe at 300 nM, and RCKr_F and RCKr_R primers at 500 nM, and RCKr_P at 80 nM. The nucleic acid input volume was 10 µL for a final reaction volume of 25 µL. Reverse transcription, amplification, and fluorescence detection were performed using an ABI 7500 FAST DX Sequence Detection System (Thermo Fisher Scientific, www.thermofisher.com/us/en/home.html) with the following cycling parameters: 50°C for 10 min, 95°C for 2 min followed by 40 cycles of 95°C for 3 s and 60°C for 40 s. Fluorescent readings were collected during the 60°C anneal/extension step.

References

1. Chung IH, Robinson LK, Stewart-Juba JJ, Dasch GA, Kato CY. Analytically sensitive *Rickettsia* species detection for laboratory diagnosis. Am J Trop Med Hyg. 2022;106:1352–7. PubMed https://doi.org/10.4269/ajtmh.21-0757

Appendix Table 1. Triplex rRT-PCR oligonucleotide primer and probe sequences

Assay	Analyte	Oligonucleotide Name	Oligonucleotide Sequence and Modifications*
Rickettsia Triplex rRT-PCR	*Rickettsia* 23S rRNA†	RCKr_F	GGTCCYACAGACTTACCAAACTCA
		RCKr_R	TCGACTATGGACCTTAGCACCCAT
		RCKR_P	VIC-CCGAATGTCGATGAGTACAGCATAGCAGAC-QSY
	R. rickettsii 23S rRNA SNP	Rrick23S_F	GCGATGAAGGACGTAATACGCT
		Rrick23S_R	TAGGTAGGTTTCCCTATTCGGA
		Rrick23S_P	Q670-CGGATCGAAGTTTATTCGCA-BHQ2
	R. typhi 23S rRNA SNP	Rtyp23S_F	CTAACGCCTCTGCTTCGCAG
		Rtyp23S_R	GAAAGACCCCGTGAACCTTTACTA
		Rtyp23S_P	6FAM-TGCACATTT/ZEN/GACTTCTAACACC-3IABkFQ

*Oligonucleotide modifications: 6FAM (6-carboxyfluorescein), VIC, Q670 (Quasar 670) are fluorescent dyes. QSY, BHQ2 (Black Hole Quencher 2), ZEN, and IABkFQ (Iowa Black Fluorescent Quencher) are nonfluorescent acceptor dyes.
†Modified from Chung et al. (*1*).

Appendix Table 2. Primer sequences, master mix, and amplification conditions for MLST

Amplification Reaction	Primer Name	Primer Sequences	Primer Reaction Concentration	Master Mix	Amplification Parameters
23S rRNA Outer RT-PCR Long Amplicon	RkAS77F2	CTTCGGGGAGTTGCGAATAA	600 nM	Superscript IV One-Step RT-PCR System	55°C for 20 min, 98°C for 2 min, 45 cycles of 98°C for 10 s, 64°C for 10 s, 72°C for 1 min, hold at 72°C for 5 min
	Rk23S1606R	GGTCATCTTTCTTCCGAAGTTACAG	600 nM		
23S rRNA Inner PCR Long Amplicon	RkAS77F2	CTTCGGGGAGTTGCGAATAA	240 nM	Q5 Hot Start, High Fidelity	98°C for 30 s, 35 cycles of 98°C for 5 s, 64°C for 20 s, 72°C for 40 s, hold at 72°C for 2 min
	Rk23S1588R	GTTACAGATGYAATTTGCCTAGTT	240 nM		
23S rRNA Outer RT-PCR Short Amplicon	Rk23S42F	AGAAGGCGATGAAGGACGTAAT	600 nM	Superscript IV One-Step RT-PCR System	55°C for 20 min, 98°C for 2 min, 45 cycles of 98°C for 10 s, 62°C for 10 s, 72°C for 30 s, hold at 72°C for 5 min
	RkPan558R	GGTACGCCGTCACAAGACAT	600 nM		
23S rRNA Inner PCR Short Amplicon	RkAS77F2	CTTCGGGGAGTTGCGAATAA	240 nM	Q5 Hot Start, High Fidelity	98°C for 30 s, 35 cycles of 98°C for 5 s, 64°C for 20 s, 72°C for 20 s, hold at 72°C for 2 min
	Rk23S517R	GCTTGTAAGCATTTGGTTTCAGAT	240 nM		

Amplification Reaction	Primer Name	Primer Sequences	Primer Reaction Concentration	Master Mix	Amplification Parameters
16S rRNA Outer RT-PCR	Rk16S53F	AGTCTTTAAGGAGGTAATCCAGC	400 nM	Superscript IV One-Step RT-PCR System	55°C for 20 min, 98°C for 2 min, 45 cycles of 98°C for 10 s, 62°C for 10 s, 72°C for 1 min, hold at 72°C for 5 min
	Rk16S1570R	AACTGACAGAATCAAACTTGAGAGT	400 nM		
16S rRNA Inner PCR	Rk16S53F	AGTCTTTAAGGAGGTAATCCAGC	200 nM	Q5 Hot Start, High Fidelity	98°C for 30 s, 35 cycles of 98°C for 5 s, 64°C for 30 s, 72°C for 30 s, hold at 72°C for 2 min
	Rk16S1556R	AACTTGAGAGTTTGATCCTGGCT	200 nM		
gltA PCR	SFGgltA71F	GTTCAGGGTCTTCGTGCATTTC	200 nM	Q5 Hot Start, High Fidelity	98°C for 1 min, 45 cycles of 98°C for 10 s, 64°C for 20 s, 72°C for 1 min, hold at 72°C for 2 min
	SFGgltA1226R	AAAGCAAGTATCGGTGAGGATG	200 nM		
ompA PCR	RR190–70	ATGGCGAATATTTCTCCAAAA	200 nM	Q5 Hot Start, High Fidelity	98°C for 1 min, 45 cycles of 98°C for 10 s, 60°C for 20 s, 72°C for 40 s, hold at 72°C for 2 min
	RR190–701	GTTCCGTTAATGGCAGCATCT	200 nM		
*ompB*1–2 Outer PCR	SFGompB65F	GAGCGATTAGAAGTTTACACGGA	200 nM	Q5 Hot Start, High Fidelity	98°C for 1 min, 45 cycles of 98°C for 10 s, 60°C for 20 s, 72°C for 1:30 min, hold at 72°C for 2 min
	SFGompB2850R	CTACTACCGATGCTAACGTAGGT	200 nM		
*ompB*1 Inner PCR	SFGompB65F	GAGCGATTAGAAGTTTACACGGA		Q5 Hot Start, High Fidelity	98°C for 1 min, 35 cycles of 98°C for 5 s, 64°C for 20 s, 72°C for 40 s, hold at 72°C for 2 min
	SFGompB1609R	GCAAGTGGTACTTCAACATGGG	200 nM		
*ompB*2 Inner PCR	SFGompB1391F	GTTAAATCTAGCACCACCTTGGA	200 nM	Q5 Hot Start, High Fidelity	98°C for 1 min, 35 cycles of 98°C for 5 s, 64°C for 20 s, 72°C for 40 s, hold at 72°C for 2 min
	SFGompB2850R	CTACTACCGATGCTAACGTAGGT	200 nM		
*ompB*3–4 Outer PCR	SFGompB2692F	CGTTAAATGTTGCATTACCTAAGAACT	200 nM	Q5 Hot Start, High Fidelity	98°C for 1 min, 45 cycles of 98°C for 10 s, 60°C for 20 s, 72°C for 1:30

Amplification Reaction	Primer Name	Primer Sequences	Primer Reaction Concentration	Master Mix	Amplification Parameters
					min, hold at 72°C for 2 min
	SFGompB5000R	AGGGTTGGTAACTGCTTCTACAG	200 nM		
*ompB*3 Inner PCR	SFGompB2692F	CGTTAAATGTTGCATTACCTAAGAACT	400 nM	Q5 Hot Start, High Fidelity	98°C for 1 min, 35 cycles of 98°C for 5 s, 64°C for 20 s, 72°C for 40 s, hold at 72°C for 2 min
	SFGompB4180R	GTTACAGGAAGTTTAGGCGGT	400 nM		
*ompB*4 Inner PCR	SFGompB3546F	CCGTTTATAACTGTACCGTCAGC	200 nM	Q5 Hot Start, High Fidelity	98°C for 1 min, 35 cycles of 98°C for 5 s, 64°C for 20 s, 72°C for 40 s, hold at 72°C for 2 min
	SFGompB5000R	AGGGTTGGTAACTGCTTCTACAG	200 nM		
sca4-1 PCR	SFGsca4–67F	TGAGAGGTTTTATGAGTAAAGACGGT	200 nM	Q5 Hot Start, High Fidelity	98°C for 1 min, 45 cycles of 98°C for 10 s, 64°C for 20 s, 72°C for 1 min, hold at 72°C for 2 min
	SFGsca4–1734R	CAATTGCTGCAGCTCTACTTGC	200 nM		
sca4-2 PCR	SFGsca4–1533F	GCTGGAGTCAATGCAGTATTAGAA	200 nM	Q5 Hot Start, High Fidelity	98°C for 1 min, 45 cycles of 98°C for 10 s, 64°C for 20 s, 72°C for 1 min, hold at 72°C for 2 min
	SFGsca4–3158R	ACAGAATACAAATCTTGATCAGCGT	200 nM		

Appendix Table 3. MLST sequencing primers

Amplicon	Primer Name	Sequence
23S rRNA	RkAS77F2	CTTCGGGGAGTTGCGAATAA
	RkAS503F	CAAATGCTTACAAGCAGTCGGA
	Rk23S517R	GCTTGTAAGCATTTGGTTTCAGAT
	Rk23S598R	GCTTGCTAGATAAACTAAGTCGC
	Rk23S1025F	GATGTGAGAAGACCAAAACAACTA
	RkAS1158R	TCTTCGGTACATGACTTGAGCC
	Rk23S1588R	GTTACAGATGYAATTTGCCTAGTT
16S rRNA	Rk16S55F	AGTCTTTAAGGAGGTAATCCAGC
	Rk16S656F	TTATGCGTTAGCTGCGAAAC
	Rk16S709R	CGATGAGTGCTAGATATCGGAA
	Rk16S1556R	AACTTGAGAGTTTGATCCTGGCT
gltA	SFGgltA71F	GTTCAGGGTCTTCGTGCATTTC
	RkgltA506F	GCATATGATGTTTGCAACGC
	RkgltA602R	CTCATGATCGGCATGTAGGA
	SFGgltA1226R	AAAGCAAGTATCGGTGAGGATG
ompA	RR190–70	ATGGCGAATATTTCTCCAAAA
	RR190–701	GTTCCGTTAATGGCAGCATCT
*ompB*1	SFGompB67F	GAGCGATTAGAAGTTTACACGGA
	RkompB1–815F	GCTAGCGTATCTAAACCGATTAC
	RkompB1–893R	CACATCAAAGTAAGAAAGGTGGT
	SFGompB1609R	GCAAGTGGTACTTCAACATGGG
*ompB*2	SFGompB1391F	GTTAAATCTAGCACCACCTTGGA
	RkompB2–1996F	ACCGTCATTAATTGTTGCGTTA

Amplicon	Primer Name	Sequence
	RkompB2–2123R	ATACCCCTGGTACAGTTTATGG
	SFGompB2850R	CTACTACCGATGCTAACGTAGGT
*ompB*3	SFGompB2692F	CGTTAAATGTTGCATTACCTAAGAACT
	RkompB3–3479F	GCACTACCGTCTAAGGTAATAGT
	RkompB3–3592R	TGCCGGTTCTATCTTTAAACTTG
	SFGompB4180R	GTTACAGGAAGTTTAGGCGGT
*ompB*4	SFGompB3546F	CCGTTTATAACTGTACCGTCAGC
	RkompB4–4214F	AATACTAATCTACCCGTACCGTC
	RkompB4–4278R	AATTTACAAGCAGGTGGTACT
	SFGompB5000R	AGGGTTGGTAACTGCTTCTACAG
sca4-1	SFGsca4–67F	TGAGAGGTTTTATGAGTAAAGACGGT
	RkSka4a758F	GCCGTATATTTCACTGCCCA
	RkSka4a856R	TCCGGTTCCGGCAAATTTTA
	SFGsca4–1734R	CAATTGCTGCAGCTCTACTTGC
sca4-2	SFGsca4–1533F	GCTGGAGTCAATGCAGTATTAGAA
	Rksca4–2322F	AGGTCCTGAATAACTAAGGCA
	Rksca4–2399R	TTAAGTGCCTCTTTCTCAGAGT
	SFGsca4–3158R	ACAGAATACAAATCTTGATCAGCGT

Appendix Table 4. *Rickettsiales* nucleic acids used for assessing analytical specificity of the *Rickettsia* sp. CA6269 Real-time PCR Assay

Organism	Strain	Source
Anaplasma phagocytophilum	NCH-1	BEI Resources
Ehrlichia chaffeensis	St. Vincent	BEI Resources
Orientia tsutsugamushi	Karp	CDC
Rickettsia africae	R8637	CDC
R. akari	Kaplan	CDC
R. amblyommatis	GAT-30V	CDC
R. asembonensis	NMRC11	CDC
R. conorii	R0362	CDC
R. felis	Baton Rouge	CDC
R. massiliae	AZT80	CDC
R. parkeri	Maculatum 20	CDC
R. prowazekii	Madrid II	CDC
R. rhipicephali	20–4164	CDC
R. sibirica	246	BEI Resources
R. tillamookensis	Tillamook 23	CDC
R. typhi	Wilmington	CDC
R. rickettsii	Hlp2	CDC
R. rickettsii	Columbia	CDC
R. rickettsii	Costa Rica	CDC
R. rickettsii	Brazil	CDC
R. rickettsii	Hauke	CDC
R. rickettsii	80JC	CDC
R. rickettsii	Coleman	CDC
R. rickettsii	Stewart	CDC
R. rickettsii	76RC	CDC
R. rickettsii	Sheila Smith	CDC
Rickettsia sp. 364D	Pierce Canyon	CDC
Rickettsia sp. 364D	Wright Peak	CDC
Rickettsia sp. 364D	Pine Mountain	CDC
Rickettsia sp. 364D	Crystal Cove	CDC
Rickettsia sp. 364D	El Moro Canyon	CDC
Rickettsia sp. 364D	Lake	CDC
Rickettsia sp. 364D	Cache Creek Ridge	CDC
Rickettsia sp. 364D	Highland Creek	CDC
Rickettsia sp. 364D	Orange	CDC
Rickettsia sp. 364D	Mt. Konocti	CDC
Rickettsia sp. 364D	364D	CDC
